# Diagnostic criteria for severe acute malnutrition and fatal outcomes in children aged 6–59 months, Nigeria

**DOI:** 10.2471/BLT.24.292143

**Published:** 2025-06-10

**Authors:** Emmanuel Grellety, Erica Simons, Mathilde Mousset, Thomas Roederer, Avilah-Phrygie Amakade-Woyengba, Sabino Malwal, Olatunji Joyce Adebayo, Bérengère Guais, Michel O Lacharité, Guyguy Manangama, Nafisa Sani Nass

**Affiliations:** a Department of Epidemiology Intervention and Training, Epicentre, 14–34 Avenue Jean Jaurès, 75019 Paris, France.; b Department of Data Science, Epicentre, Paris, France.; c Bordeaux School of Public Health, University of Bordeaux, Bordeaux, France.; d Department of Emergency, Médecins Sans Frontières Operational Centre Paris, Katsina, Nigeria.; e Department of Emergency, Médecins Sans Frontières Operational Centre Paris, Paris, France.; f State Primary Health Care Agency, Katsina, Nigeria.

## Abstract

**Objective:**

To determine factors associated with inpatient death among a cohort of children aged 6–59 months with severe acute malnutrition in north-western Nigeria.

**Methods:**

Our observational study used routine programmatic data of all children aged 6–59 months admitted to two inpatient facilities in Katsina State with severe acute malnutrition in 2022. We assessed nutritional status at admission by weight-for-height z-score (WHZ), mid-upper-arm circumference (MUAC) and bilateral nutritional oedema using World Health Organization definitions. We used Cox-proportional hazard models to identify predictors of mortality, with and without adjustment for sex, age group, nutritional status at admission, major clinical complications and comorbidities.

**Findings:**

Of 12 771 children included in the analysis, we observed an overall inpatient mortality of 8.4%. Compared with children admitted by the MUAC criterion alone, we noted that children admitted by the WHZ criterion alone had twice the risk of death; children admitted with kwashiorkor and low WHZ had more than four times the risk. Older children with marasmus had a higher risk of death than younger children (adjusted hazard ratio: 1.74; 95% confidence interval: 1.50–2.03). We did not observe any significant association between stunting and mortality. Our findings were not altered by any of the complications or comorbidities recorded.

**Conclusion:**

Children with a low WHZ at admission have a higher risk of death than those with a low MUAC, and should be subject to special considerations when associated with oedema. MUAC alone is an insufficient criterion to identify all the children at risk of death from malnutrition.

## Résumé

**Objectif:**

Déterminer les facteurs associés au décès dans une cohorte d’enfants hospitalisés âgés de 6 à 59 mois souffrant de malnutrition aiguë sévère dans le nord-ouest du Nigéria.

**Méthodes:**

Notre étude observationnelle a utilisé les données programmatiques de routine de tous les enfants âgés de 6 à 59 mois admis en 2022 pour malnutrition aiguë sévère dans deux établissements hospitaliers de l’État de Katsina. Nous avons évalué l’état nutritionnel à l’admission à partir de l’indice poids pour taille exprimé en Z-score (PTZ), le périmètre brachial (PB) et les œdèmes nutritionnels bilatéraux selon les définitions de l’Organisation mondiale de la Santé. Nous avons appliqué des modèles à risques proportionnels de Cox pour identifier les facteurs prédictifs de mortalité, avec et sans ajustement pour le sexe, le groupe d’âge, l’état nutritionnel à l’admission, les principales complications cliniques et les comorbidités.

**Résultats:**

Sur les 12 771 enfants inclus dans l’analyse, nous avons observé une mortalité hospitalière globale de 8,4%. Comparés aux enfants admis selon le seul critère du PB, nous avons remarqué que les enfants admis selon le seul critère du PTZ présentaient un risque de décès deux fois plus élevé; les enfants admis avec un kwashiorkor et un faible PTZ présentaient un risque plus de quatre fois supérieur. Les enfants marasmiques plus âgés présentaient un risque de décès plus élevé que les enfants plus jeunes (rapport de risque ajusté: 1,74; intervalle de confiance à 95%: 1,50–2,03). Nous n’avons pas observé d’association significative entre le retard de croissance et la mortalité. Nos résultats n’ont pas été modifiés par les complications ou les comorbidités enregistrées.

**Conclusion:**

Les enfants avec un faible PTZ à l’admission présentent un risque de décès plus élevé que ceux avec un faible PB et devraient faire l’objet d’attentions particulières lorsqu’associé à des œdèmes. Le PB seul est un critère insuffisant pour identifier tous les enfants à risque de décès lié à la malnutrition.

## Resumen

**Objetivo:**

Determinar los factores asociados a la mortalidad intrahospitalaria en una cohorte de niños de 6 a 59 meses con desnutrición aguda grave en el noroeste de Nigeria.

**Métodos:**

Este estudio observacional utilizó datos rutinarios de programa correspondientes a todos los niños de 6 a 59 meses admitidos en 2022 por desnutrición aguda grave en dos centros de hospitalización del estado de Katsina. Se evaluó el estado nutricional al ingreso mediante la puntuación Z peso/talla (WHZ, por sus siglas en inglés), la circunferencia media del brazo (MUAC, por sus siglas en inglés) y la presencia de edemas nutricionales bilaterales, según las definiciones de la Organización Mundial de la Salud. Se emplearon modelos de riesgos proporcionales de Cox para identificar predictores de mortalidad, con y sin ajuste por sexo, grupo etario, estado nutricional al ingreso, principales complicaciones clínicas y comorbilidades.

**Resultados:**

De los 12 771 niños incluidos en el análisis, se observó una mortalidad intrahospitalaria global del 8,4%. En comparación con los niños admitidos únicamente por el criterio de MUAC, se observó que los admitidos únicamente por el criterio de WHZ presentaban el doble de riesgo de muerte; los niños admitidos con kwashiorkor y puntuación WHZ baja presentaban más de cuatro veces el riesgo. Los niños marasmáticos de mayor edad presentaban un mayor riesgo de fallecimiento que los más pequeños (razón de riesgo ajustada: 1,74; intervalo de confianza del 95%: 1,50-2,03). No se observó ninguna asociación significativa entre retraso del crecimiento y mortalidad. Los resultados no se vieron modificados por ninguna de las complicaciones ni comorbilidades registradas.

**Conclusión:**

Los niños con puntuación WHZ baja al ingreso presentan un mayor riesgo de muerte que aquellos con MUAC bajo. Además, deben ser objeto de consideraciones especiales cuando se asocia edema. El uso exclusivo de MUAC es un criterio insuficiente para identificar a todos los niños con riesgo de fallecer por desnutrición.

## ملخص

الغرض تحديد العوامل المرتبطة بوفيات المرضى الداخليين بين مجموعة من الأطفال الذين تتراوح أعمارهم بين 6 و59 شهرًا، والذين يعانون من سوء التغذية الحاد الشديد في شمال غرب نيجيريا.

الطريقة استعانت الدراسة الرصدية لدينا على بيانات برمجية روتينية لجميع الأطفال الذين تتراوح أعمارهم بين 6 و59 شهرًا، والذين تم إلحاقهم في منشأتين للمرضى الداخليين في ولاية كاتسينا عام 2022، والذين يعانون من سوء التغذية الحاد الشديد. قمنا بتقييم حالتهم الغذائية عند الدخول باستخدام تقييم Z لنسبة الوزن إلى الطول (WHZ)، ومحيط منتصف الذراع (MUAC)، والوذمة الغذائية الثنائية، وذلك باستخدام تعريفات منظمة الصحة العالمية. وقمنا بالاستعانة بنماذج المخاطر النسبية Cox لتحديد عوامل التنبؤ بالوفيات، مع أو دون تعديلات على الجنس، والفئة العمرية، والحالة الغذائية عند الدخول، والمضاعفات الإكلينيكية الرئيسية، والأمراض المصاحبة.

النتائج من بين 12771 طفلاً مشمولين في التحليل، لاحظنا معدلاً إجماليًا لوفيات المرضى الداخليين بنسبة %8.4. وبالمقارنة مع الأطفال الذين تم إدخالهم وفقًا لمعيار MUAC فقط، لاحظنا أن الأطفال الذين تم إلحاقهم وفقًا لمعيار WHZ فقط، لديهم خطر مضاعف للوفاة؛ بينما الأطفال الذين تم إلحاقهم وهم يعانون من مرض كواشيوركور وتقييم WHZ منخفض، كان لديهم أكثر من أربعة أضعاف للخطر. وكان لدى الأطفال الأكبر سنًا المصابين بالهزال، خطر وفاة أعلى من الأطفال الأصغر سنًا (نسبة المخاطرة المعدلة: 1.74؛ وبفاصل ثقة مقداره %95: 1.50 إلى 2.03). ولم نلاحظ أي ارتباط ملحوظ بين التقزم والوفاة. ولم تتغير نتائجنا بسبب أي من المضاعفات أو الأمراض المصاحبة المسجلة.

الاستنتاج الأطفال الذين يعانون من تقييم WHZ منخفض عند دخولهم المستشفى، كانوا أكثر عرضة لخطر الوفاة من أولئك الذين يعانون من معيار MUAC منخفض، ويجب أن يخضعوا لاعتبارات خاصة عند ارتباطهم بالوذمة. يُعد MUAC وحده معيارًا غير كاف لتحديد جميع الأطفال المعرضين لخطر الوفاة بسبب سوء التغذية.

## 摘要

**目的:**

旨在确定在尼日利亚西北部因重度急性营养不良而住院治疗的 6-59 个月大孩子的死亡相关因素。

**方法:**

我们在观察性研究中使用了 2022 年在卡齐纳州因重度急性营养不良而入住过两家医疗机构的所有 6-59 个月大孩子的常规治疗方案数据。根据世界卫生组织定义的身长别体重 Z 评分 (WHZ)、上臂围 (MUAC) 标准和营养不良性双侧水肿标准，我们评估了入院时的营养状况。在根据和不根据性别、年龄组、入院时的营养状况以及主要临床并发症和合并症进行调整的情况下，我们使用 Cox 比例风险回归模型确定了死亡率相关预测因素。

**结果:**

据我们观察，在被我们纳入分析范畴的 12,771 名孩子中，住院患者总死亡率为 8.4%。我们发现，与仅按照 MUAC 筛查标准被允许住院接受治疗的孩子相比，仅按照 WHZ 筛查标准被允许住院接受治疗的孩子的死亡风险是前者的两倍；而因夸希奥科病和 WHZ 评分低而被允许住院接受治疗的孩子的风险更是前者的 4 倍以上。在这些消瘦的孩子中，年长者比年幼者的死亡风险更高（调整后风险比：1.74；95% 置信区间：1.50-2.03）。我们并未发现发育迟缓和死亡率有何重大关联。将记录的任何并发症或合并症纳入考虑范围后，我们的研究结果也并未发生改变。

**结论:**

与入院时 MUAC 测量数值低的孩子相比，WHZ 评分低的孩子的死亡风险更高，如果发现这类孩子还存在水肿现象，则应特别注意。仅凭借 MUAC 筛查标准不足以确定所有孩子是否有因营养不良而致死的风险。

## Резюме

**Цель:**

Определить факторы, связанные со смертями в стационарных лечебных учреждениях северо-западной части Нигерии среди когорты детей, страдающих от тяжелого острого недоедания, в возрасте от 6 до 59 месяцев.

**Методы:**

В этом обсервационном исследовании изучались регулярные, планомерно получаемые данные по всем детям в возрасте от 6 до 59 месяцев, которые были госпитализированы в два стационарных учреждения штата Кацина в 2022 году по причине острого тяжелого недоедания. Авторы оценивали нутритивный статус детей при поступлении по шкале соотношения веса и роста Z (weight-for-height Z-score, WHZ), окружности плеча на середине высоты (mid-upper-arm circumference, MUAC) и наличию двустороннего алиментарного отека по определениям Всемирной организации здравоохранения. Использовалась пропорциональная модель рисков Кокса для выявления прогностических факторов смертности как с поправкой на пол, возраст, алиментарный статус при поступлении, основные клинические осложнения и сопутствующие заболевания, так и без нее.

**Результаты:**

Анализ данных 12 771 ребенка показал, что общая смертность в стационаре составила 8,4%. В сравнении с детьми, которые поступили только по критерию MUAC, отмечалось следующее: при поступлении только по критерию WHZ риск смерти ребенка был вдвое выше, при этом у детей, поступивших с квашиоркором и низким баллом по шкале WHZ, такой риск увеличивался более чем в четыре раза. Истощенные дети старшего возраста имели более высокий риск смерти, нежели младшие (скорректированное отношение рисков: 1,74; 95%-й ДИ: 1,50–2,03). Значимой связи между задержкой роста и смертностью не наблюдалось. На результаты исследования не повлияли какие бы то ни было осложнения или зафиксированные сопутствующие заболевания.

**Вывод:**

Дети с низким баллом по шкале WHZ при поступлении имели более высокий риск смерти, чем при низком балле MUAC. Следует также обращать особое внимание на состояние детей, если к этому симптому присоединяется алиментарный отек. Сам по себе MUAC не является достаточным критерием для выявления всех детей, которые подвержены риску смерти от недоедания.

## Introduction

Each year severe acute malnutrition contributes to millions of deaths among children aged 6–59 months. These estimates are based on weight-for-height z-scores (WHZ).^1^ If children with a low mid-upper-arm circumference (MUAC) and nutritional oedema (kwashiorkor) are added, the prevalence estimate doubles;^2^^,^^3^ when incidence instead of prevalence is considered, the burden increases dramatically.^4^ Regardless of the exact numbers, severe acute malnutrition is a major concern.

The World Health Organization (WHO) defines severe acute malnutrition as any combination of WHZ less than −3Z, mid-upper-arm circumference less than 115 mm and/or bilateral nutritional oedema.^5^ Affected children have a higher risk of death by a factor of approximately nine compared with healthy children.^6^ Children without clinical complications can be managed in the community through outpatient therapeutic programmes; however, children who fail an appetite test, have severe oedema, medical complications or clinical danger signs are categorized as having complicated severe acute malnutrition and are initially managed in inpatient facilities.^7^

In sub-Saharan Africa, 10–40% of children with severe acute malnutrition admitted for inpatient care die.^8^ WHO predicts that the mortality could be reduced to less than 5% if current WHO treatment guidelines are followed.^9^ However, few studies have reported on factors associated with inpatient mortality from severe acute malnutrition. A review of 19 such studies since 2000 only had 400 patients per study.^10^ As well as having insufficient power or using outdated guidelines and standards, the results of these studies had not been adjusted for important confounding factors including complications, comorbidities and nutritional oedema.^3^^,^^11^ Early identification of prognostic factors, referral to inpatient facilities and risk stratification at admission could reduce deaths of affected children.

Katsina State in north-western Nigeria has 34 local government areas with an estimated population of 10.3 million.^12^ The region has recently faced high levels of banditry and is the most food insecure in Nigeria; recent surveys show alarming levels of malnutrition classified under the Integrated Food Security Phase Classification for Acute Malnutrition as Phase 3 (serious) to Phase 4 (critical).^13^^–^^15^ Since 2021, *Médecins Sans Frontières* (MSF) Operational Centre Paris with the Katsina State Ministry of Health has managed acute malnutrition in the local government areas of Jibia, Katsina and Mashi, admitting the largest cohort of children with severe acute malnutrition ever treated by MSF.^16^ Among all patients treated in the MSF therapeutic programmes, almost all deaths occurred in children admitted to one of the inpatient facilities. To provide insights into the mechanisms underlying inpatient mortality, we examine the factors associated with death among children with severe acute malnutrition.

## Methods

We collected clinical and anthropometric data from all patients aged 6–59 months admitted to the two inpatient MSF facilities in Katsina town from 1 January to 31 December 2022. Treatment in these inpatient facilities followed WHO guidelines and the current national protocol.^9^^,^^17^

Data encoders routinely entered data from patient files and registers into an Excel (Microsoft, Redmond, United States of America) line list to facilitate supervision, audit and early detection of anomalous mortality. Staff entered data at admission and completed each patient’s details at or shortly after discharge. We de-identified data before compiling weekly analyses.

**Fig. 1:**
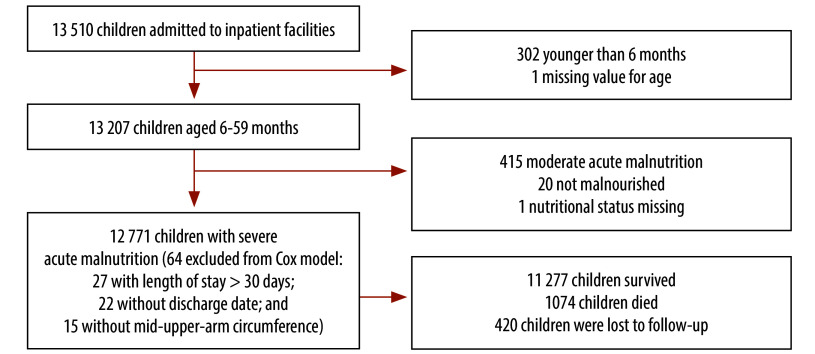
Flowchart of children aged 6–59 months with severe acute malnutrition admitted to inpatient facilities and children included in analyses, Katsina, Nigeria 2022

We excluded children that did not meet the criteria for severe acute malnutrition, or had implausible or missing weight, height, MUAC, oedema or outcome, from our analysis (Fig. 1).

### Statistical analysis

Our primary outcome was all-cause in-hospital mortality. Children who defaulted (i.e. were lost to follow-up) were included in the analysis. There were no transfers to other institutions.

We defined our independent variables as age group (6–23 and 24–59 months); sex; local government area of residence (either supported or not supported by MSF); type of admission (direct admission or transfer from an outpatient therapeutic programme); weight; height; MUAC; WHZ; height-for-age z-score (HAZ); nutritional status; nutritional grade (oedema in the feet, hands and face classified as grades 1, 2 and 3, respectively); major clinical complications; and common comorbidities (including a rapid malaria test at admission). Health workers diagnosed dehydration and sepsis from clinical signs alone, respiratory infection by tachypnoea alone, and hypoglycaemia mostly by clinical signs and occasionally blood glucose. We assessed nutritional status based on current WHO definitions^5^ and, to avoid Simpson’s paradox (an extreme form of confounding where the results of a comparison can be reversed, making a less important variable appear dominant),^3^^,^^18^^,^^19^ we classified children into one of the categories defined in Table 1.

**Table 1:** Classification of nutritional status of children aged 6–59 months with severe acute malnutrition admitted to two inpatient facilities in Katsina, Nigeria, 2022

Nutritional status	Oedema	MUAC (mm)	WHZa
MUAC only	No	< 115	≥ −3Z
WHZ only	No	≥ 115	< −3Z
WHZ and MUAC	No	< 115	< −3Z
Oedema only	Yes	≥ 115	≥ −3Z
Oedema and MUAC	Yes	< 115	≥ −3Z
Oedema and WHZ	Yes	≥ 115	< −3Z
Oedema, WHZ and MUAC	Yes	< 115	< −3Z

MUAC: mid-upper-arm circumference; WHZ: weight-for-height z-score.

We calculated distributions of patients and deaths over each level of the specified variables. Subsequently, we performed Pearson’s χ^2^ tests (*P*-value < 0.05) to examine whether the distributions of deaths differed across levels, in relation to case fatality rates.

We then ran univariable Kaplan–Meier models comparing mean or median survival times for each variable. Variables with a *P*-value of less than 0.05 were retained and paired for bivariable models to test for the presence of one-way interactions. We included all significant covariates and interactions in a full multivariable analysis, both with and without adjustment for the potential confounders of sex, age group, nutritional status at admission, major clinical complications, comorbidities and their relevant interactions. Given the persistence of the interaction between age group and nutritional status groups in the full model (*P*-value < 0.05), we also present stratified multivariable analyses for children with marasmus (that is, non-oedematous malnutrition) and kwashiorkor, disaggregating these distinct malnutrition forms.

We report both crude and adjusted hazard ratios (HR and aHR) with 95% confidence intervals (CI). A comparison of aHR with HR shows the magnitude of association between death and covariates. We assessed the proportional hazard assumption based on Schoenfeld residuals test (both global and scaled) and log-minus-log graphs. We considered covariates with a *P*-value of less than 0.05 to be statistically significant predictors of death in children with severe acute malnutrition.

We conducted a sensitivity analysis to determine whether results varied when children recorded as defaulters were considered as deaths.

We analysed all data using R software, version 4.2.0 (R Foundation for Statistical Computing, Vienna, Austria).

### Ethics

Our research fulfilled the exemption criteria set by the MSF Ethics Review Board for retrospective analyses of routinely collected clinical data, and was conducted with the permission of the Medical Director of MSF Operation Centre Paris. The Katsina State Ministry of Health additionally approved the secondary use of routinely collected programmatic data for our analysis and publication. Informed consent was not required as the data were collected for monitoring and evaluation purposes within the MSF-supported nutrition programme.

## Results

Our initial analysis included 12 771 children; an anthropometric measurement (MUAC) had not been recorded for 15 children (0.1%) and a further 49 children were excluded from the Cox analysis (0.4%; Fig. 1).

**Fig. 2:**
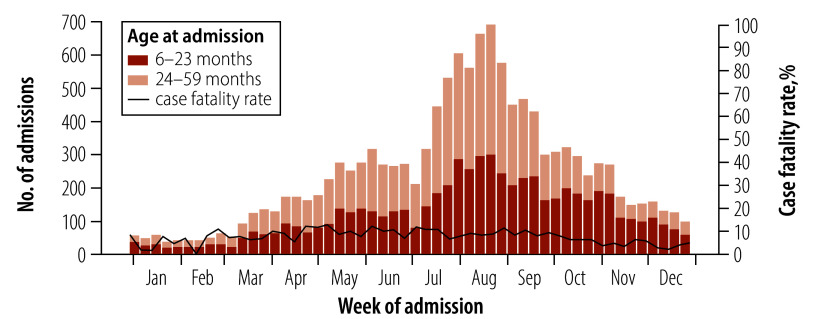
Number of children aged 6–59 months with severe acute malnutrition admitted to, and weekly case fatality rate within, inpatient facilities in Katsina, Nigeria, 2022

We observed that the case fatality rate remained steady throughout the year at about 8.4% (1074/12 771; monthly range: 3.8–10.8), despite the seasonality in admissions (Fig. 2). A slightly higher proportion of older children were admitted during the peak. A total of 5 826/12 771 (45.6%) patients were direct admissions to the inpatient facility, mostly from local government areas not supported by MSF (9012; 70.6%). Table 2 shows that children aged 6 to 23 months were 50.1% (6396/12 771) of those admitted. 49.0% of children admitted were girls (6258/12 771). Of the deaths, 792/1074 (73.7%) were from local government areas not supported by MSF. We observed that the older children had a much higher risk of death than the younger children (aged 24–59 months: 648; 60.3%), and girls accounted for 579/1074 (53.9%) of the deaths.

**Table 2:** General characteristics and case-fatality rates of children aged 6–59 months with severe acute malnutrition admitted to two inpatient facilities in Katsina, Nigeria, 2022

Characteristic	No. children (%)			Case fatality rate (%)	*P* ^a^
	With severe acute malnutrition (*n* = 12 771)	Mortality (*n* = 1 074)			
**Type of admission**					0.7
Direct	5 826 (45.6)	496 (46.2)		8.5	
Transfer	6 945 (54.4)	578 (53.8)		8.3	
**Local government area**					0.017
Not supported by MSF	9 012 (70.6)	792 (73.7)		8.8	
Supported by MSF	3 759 (29.4)	282 (26.3)		7.5	
**Sex**					< 0.001
Female	6 258 (49.0)	579 (53.9)		9.3	
Male	6 513 (51.0)	495 (46.1)		7.6	
**Age, months**					< 0.001
6–23	6 396 (50.1)	426 (39.7)		6.7	
24–59	6 375 (49.9)	648 (60.3)		10.2	
**HAZ at admission**					0.32
≥ −2Z (not stunted)	1 474 (11.5)	134 (12.5)		9.1	
< −2Z (stunted)	11 297 (88.5)	940 (87.5)		8.3	
**MUAC at admission (mm)^b^**					0.003
< 110	6 973 (54.6)	642 (59.8)		9.2	
110 to < 115	2 628 (20.6)	186 (17.3)		7.1	
115 to < 125	2 368 (18.5)	186 (17.3)		7.9	
≥ 125	787 (6.2)	59 (5.5)		7.5	
Missing	15 (0.1)	1 (0.1)		6.7	
**WHZ at admission**					< 0.001
< −4Z	7 583 (59.4)	812 (75.6)		10.7	
−4 to < −3Z	3 316 (26.0)	184 (17.1)		5.5	
−3 to < −2Z	1 030 (8.1)	45 (4.2)		4.4	
≥ −2Z	842 (6.6)	33 (3.1)		3.9	
**Oedema grades at admission**					< 0.001
Grade 1 or 2	1 660 (13.0)	182 (16.9)		11.0	
Grade 3	2 100 (16.4)	203 (18.9)		9.7	
Missing	6 (0.0)	2 (0.2)		33.3	
**Nutritional status at admission^c^**					< 0.001
MUAC only	479 (3.8)	14 (1.3)		2.9	
WHZ only	1 531 (12.0)	98 (9.1)		6.4	
WHZ and MUAC	6 995 (54.8)	575 (53.5)		8.2	
Oedema	1 104 (8.6)	51 (4.7)		4.6	
Oedema and MUAC	289 (2.3)	13 (1.2)		4.5	
Oedema and WHZ	535 (4.2)	97 (9.0)		18.1	
Oedema, WHZ and MUAC	1 838 (14.4)	226 (21.0)		12.3	
**Major clinical complications**					< 0.001
None (failed appetite test)	3 866 (30.3)	314 (29.2)		8.1	
Moderate or severe dehydration	1 279 (10.0)	137 (12.8)		10.7	
Fever (temperature > 38.5 °C)	6 204 (48.6)	399 (37.2)		6.4	
Hypoglycaemia	639 (5.0)	143 (13.3)		22.4	
Other	783 (6.1)	81 (7.5)		10.3	
**Major comorbidities**					< 0.001
None (failed appetite test)	669 (5.2)	53 (4.9)		7.9	
Anaemia	339 (2.7)	27 (2.5)		8.0	
Acute respiratory infection	405 (3.2)	45 (4.2)		11.1	
Chronic diarrhoea	2 938 (23.0)	209 (19.5)		7.1	
Acute watery diarrhoea	1 289 (10.1)	76 (7.1)		5.9	
Malaria	2 761 (21.6)	167 (15.5)		6.0	
Measles	606 (4.7)	57 (5.3)		9.4	
Sepsis	2 485 (19.5)	328 (30.5)		13.2	
Other	1 279 (10.0)	112 (10.4)		8.8	

MSF: *Médecins Sans Frontières*; HAZ: height-for-age z-score; MUAC: mid-upper-arm circumference; WHZ: weight-for-height z-score.

^a^ We calculated *P*-values using Pearson *χ^2^*-test.

^b^ A MUAC measurement at admission was not recorded for 15 children (0.1%).

^c^ See Table 1 for definitions of the different categories of nutritional status.

We noted that 11 297/12 771 (88.5%) of the admitted patients were stunted (HAZ < −2Z) but no significant association was observed between stunting, often interpreted as chronic malnutrition, and mortality. We did not consider HAZ in any of the models explaining variations in mortality (Table 2).

We observed that many patients had very severe acute malnutrition: the majority (6973; 54.6%) had a MUAC of less than 110 mm and a WHZ of less than −4Z (7583; 59.4%), both of which corresponded to the highest case fatality rates. Such severity explains why 6995 (54.8%) of the children met the admission criteria for both MUAC and WHZ. Significantly different case fatality rates were observed by admission criterion. The lowest case fatality rate (2.9%; 14/479) was observed among children admitted by the MUAC criterion alone; those with only a low WHZ had more than double this mortality (6.4%; 98/1531), and those fulfilling both admission criteria had an even higher mortality (8.2%; 575/6995). We noted that patients with only oedema had a similar mortality (4.6%; 51/1104) to those with oedema and a low MUAC (4.5%; 13/289). Our findings reveal that children with oedema and with a low WHZ had the highest mortality of all severe acute malnutrition groups (18.1%; 97/535; Table 2).

Of all clinical complications considered, we observed that a diagnosis of hypoglycaemia was associated with the highest risk of death (22.4%; 143/639). Children with dehydration had a slightly increased mortality (10.7%; 137/1279), whereas patients with fever had a lower mortality rate (6.4%; 399/6204). Among comorbidities, we noted a lower risk of death for both chronic diarrhoea (7.1%; 209/2938) and acute diarrhoea (5.9%; 76/1289). Our results show that clinically diagnosed sepsis (13.2%; 328/2485) and rapid respiration (11.1%; 45/405) were associated significantly with mortality (Table 2).

Both the unadjusted and adjusted Cox regression models found several factors significantly associated with the probability of dying in children with marasmus (Table 3). Patients diagnosed with severe acute malnutrition based only on the WHZ criterion had about double the risk of death compared to those diagnosed using only the MUAC criterion (aHR: 1.93; 95% CI: 1.10–3.39); this result was robust after adjustment of the model. Older children had a higher risk of death than younger children (aHR: 1.74; 95% CI: 1.50–2.03) and boys a lower risk than girls (aHR: 0.82; 95% CI: 0.71–0.95). Of the complications tested, only fever, hypoglycaemia and other had a significant association with mortality. We observed that sepsis becomes significant (aHR: 1.62; 95% CI: 1.06–2.47) after adjustment for the other variables.

**Table 3:** Cox regression analysis for predictors of mortality in children aged 6–59 months with marasmus, kwashiorkor and severe acute malnutrition admitted to two inpatient facilities in Katsina, Nigeria, 2022

Predictor	With marasmus^a^			With kwashiorkor^b^			With severe acute malnutrition (both marasmus and kwashiorkor)	
	Crude HR (95% CI)	aHR (95% CI)^c^		Crude HR (95% CI)	aHR (95% CI)^c^		Crude HR (95% CI)	aHR (95% CI)^c^
**Type of admission **								
Direct	Reference	–		Reference	–		Reference	–
Transfer	0.97 (0.84–1.13)	–		0.82 (0.67–1.01)	–		0.93 (0.82–1.05)	–
**Local government area**								
Not supported by MSF	Reference	–		Reference	–		Reference	–
Supported by MSF	0.93 (0.79–1.09)	–		1 (0.78–1.27)	–		0.93 (0.82–1.07)	–
**Sex**								
Female	Reference	Reference		Reference	Reference		Reference	Reference
Male	0.81 (0.70–0.94)	0.82 (0.71–0.95)		0.88 (0.72–1.08)	0.95 (0.78–1.17)		0.84 (0.74–0.95)	0.87 (0.77–0.98)
**Age, months**								
6–23	Reference	Reference		Reference	Reference		Reference	Reference
24–59	1.9 (1.63–2.20)	1.74 (1.50–2.03)		0.83 (0.66–1.04)	0.92 (0.73–1.16)		1.52 (1.35–1.72)	1.89 (0.65–5.44)
**Nutritional status at admission^d^**								
MUAC only	Reference	Reference		NA	NA		Reference	Reference
WHZ only	2.14 (1.23–3.75)	1.93 (1.10–3.39)		NA	NA		2.13 (1.22–3.73)	1.94 (0.91–4.15)
WHZ and MUAC	2.34 (1.38–3.98)	2.07 (1.22–3.53)		NA	NA		2.32 (1.36–3.94)	2.13 (1.06–4.31)
Oedema	NA	NA		Reference	Reference		1.23 (0.68–2.23)	1.82 (0.70–4.72)
Oedema and MUAC	NA	NA		0.98 (0.53–1.80)	0.91 (0.49–1.67)		1.21 (0.57–2.56)	2.26 (0.60–8.53)
Oedema and WHZ	NA	NA		3.64 (2.58–5.12)	3.31 (2.35–4.68)		4.5 (2.57–7.89)	5.71 (2.59–12.6)
Oedema, WHZ and MUAC	NA	NA		2.45 (1.81–3.33)	2.07 (1.52–2.83)		3.04 (1.77–5.21)	3.37 (1.61–7.05)
**Major clinical complications **								
None (failed appetite test)	Reference	Reference		Reference	Reference		Reference	Reference
Moderate or severe dehydration	1.17 (0.92–1.49)	1.18 (0.92–1.52)		1.5 (1.05–2.15)	1.49 (1.03–2.15)		1.26 (1.03–1.53)	1.28 (1.04–1.57)
Fever (temperature > 38.5 °C)	0.65 (0.54–0.78)	0.73 (0.60–0.88)		1.02 (0.80–1.32)	1.08 (0.82–1.41)		0.76 (0.65–0.88)	0.83 (0.71–0.98)
Hypoglycaemia	2.57 (1.99–3.32)	2.55 (1.96–3.32)		2.62 (1.91–3.60)	2.83 (2.04–3.93)		2.57 (2.11–3.13)	2.61 (2.13–3.20)
Other	1.35 (0.99–1.84)	1.38 (1.00–1.90)		1.19 (0.79–1.78)	1.19 (0.78–1.81)		1.27 (1.00–1.62)	1.28 (0.99–1.65)
**Major comorbidities**								
None (failed appetite test)	Reference	Reference		Reference	Reference		Reference	Reference
Anaemia	0.85 (0.45–1.62)	1.12 (0.59–2.15)		1.39 (0.71–2.73)	1.45 (0.73–2.87)		1.08 (0.68–1.72)	1.32 (0.83–2.11)
Acute respiratory infection	1.12 (0.66–1.90)	1.34 (0.78–2.31)		2.14 (1.14–4.00)	2.32 (1.23–4.38)		1.44 (0.97–2.14)	1.7 (1.13–2.55)
Chronic diarrhoea	0.77 (0.50–1.18)	0.99 (0.65–1.53)		1.04 (0.67–1.61)	0.98 (0.63–1.53)		0.9 (0.67–1.22)	1.05 (0.77–1.42)
Acute watery diarrhoea	0.68 (0.42–1.08)	0.99 (0.61–1.59)		0.99 (0.54–1.81)	0.96 (0.52–1.78)		0.79 (0.56–1.13)	1.04 (0.73–1.50)
Malaria	0.72 (0.47–1.11)	0.96 (0.61–1.49)		1.05 (0.66–1.67)	1.16 (0.72–1.86)		0.86 (0.63–1.17)	1.07 (0.78–1.48)
Measles	1.17 (0.73–1.90)	1.40 (0.86–2.29)		2.29 (1.00–5.24)	2.27 (0.98–5.24)		1.41 (0.97–2.05)	1.66 (1.13–2.45)
Sepsis	1.27 (0.84–1.93)	1.62 (1.06–2.47)		2.06 (1.37–3.11)	1.97 (1.29–2.99)		1.59 (1.19–2.13)	1.84 (1.36–2.47)
Other	0.86 (0.54–1.36)	1.12 (0.70–1.80)		1.35 (0.85–2.14)	1.34 (0.83–2.15)		1.08 (0.78–1.50)	1.27 (0.91–1.77)

aHR: adjusted hazard ratio; CI: confidence interval; HR: hazard ratio; MSF: *Médecins Sans Frontières*; MUAC: mid-upper-arm circumference; WHZ: weight-for-height z-score.

^a^ Marasmus is characterized by non-oedematous severe malnutrition.

^b^ Kwashiorkor is a form of severe malnutrition causing oedema.

^c^ Hazard ratio adjusted for sex, age group, nutritional status at admission, major clinical complications and comorbidities.

^d^ See Table 1 for definitions of the different categories of nutritional status.

We noted that dehydration, diarrhoea and acute respiratory infection were not associated with mortality among children with marasmus. The same model for children with oedema found slightly different results; sex and age were not associated with the risk of death (Table 3). Low MUAC did not significantly increase the risk of death in children with oedema (aHR: 0.91; 95% CI: 0.49–1.67), but low WHZ increased the hazard ratio massively (aHR: 3.31; 95% CI: 2.35–4.68). In contrast to the children with marasmus, in children with oedema dehydration (aHR: 1.49; 95% CI: 1.03–2.15) and acute respiratory infection (aHR: 2.32; 95% CI: 1.23–4.38) increase the risk of death, but not chronic diarrhoea (aHR: 0.98; 95% CI: 0.63–1.53) or acute diarrhoea (aHR: 0.96; 95% CI: 0.52–1.78). As with marasmus, hypoglycaemia (aHR: 2.83; 95% CI: 2.04–3.93) and sepsis (aHR: 1.97; 95% 1.29–2.99) increase the risk of death for children with oedema.

**Fig. 3:**
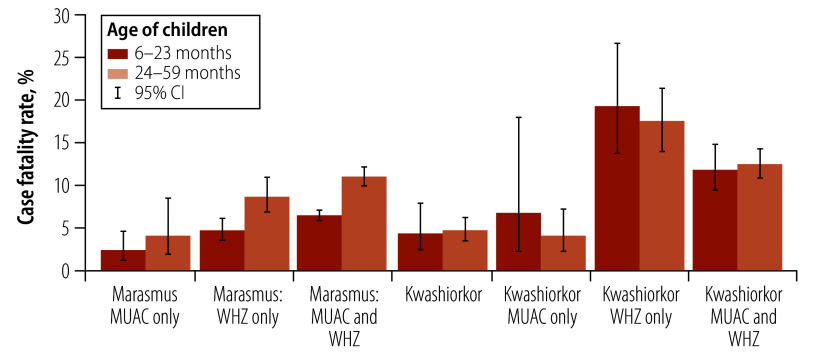
Case fatality rates of children aged 6–59 months with severe acute malnutrition by diagnostic criteria and age group admitted to inpatient facilities in Katsina, Nigeria, 2022

CI: confidence interval; MUAC: mid-upper-arm circumference; WHZ: weight-for-height z-score.

Notes: Marasmus is characterized by non-oedematous severe malnutrition. Kwashiorkor is a form of severe malnutrition causing oedema.

The full Cox regression model combining both children with marasmus and oedema broadly confirmed the results from the stratified analyses for marasmus and kwashiorkor (Table 3). Those variables that were significant either in children with marasmus and oedema separately were generally also significant in the combined data set. One exception is age, which is not significant in the full model but has an interaction with nutritional status. From the analysis of the different nutritional groups (Fig. 3), children with marasmus, but not children with oedema, have an age effect.

We ran the same model assuming that all children lost to follow-up (420 children; 3.3%) had died and found the outcome and conclusions to be unaffected. The Kaplan–Meier survival curves showed that the deaths occurred regularly throughout admission to the inpatient facility and were not clustered shortly after admission (available in online repository).^20^

## Discussion

Our retrospective analysis was based on the line list used for close surveillance and routine weekly audits of the clinical programme. All children were treated according to a standardized protocol, with no experimental interventions, additional sampling or selective inclusion beyond fulfilling the WHO-recognized diagnostic criteria. The incidence of mortality remained approximately stable, although admissions increased tenfold indicating that the protocol was implemented consistently throughout the year. 

We observed that the proportions of patients in each of our seven anthropometric groups are different from the proportions found in community surveys of this region, in which a low WHZ is more prevalent than a low mid-upper-arm circumference^14^^,^^15^^,^^21^ and few children fulfil both criteria. This difference is because the ascertainment of children at the community level was by a mid-upper-arm circumference of less than 120 mm; the children treated in the community therefore had a milder illness and a very low case fatality rate, and diagnostic proportions were closer to those found in community surveys. Overall, the combined mortality rate from severe acute malnutrition among inpatients and outpatients was 1.5% (1 130/77 244), which is well below the WHO recommended target threshold of 5%.

Our results confirm and extend the results of previous research,^19^ which analysed 14 935 inpatients with severe acute malnutrition from 17 African countries. These data showed that mortality was higher among children meeting only the WHZ criterion than those meeting only the mid-upper-arm circumference criterion. However, this study had several limitations as the researchers included historical data collected long before the publication of the current WHO admission criteria; included data from multiple inpatient facilities and agencies that were following many different (and sometimes outdated) protocols; and did not provide any information on complications or comorbidities.^19^

Our results are also consistent with data from almost 10 000 children treated for severe acute malnutrition in the Democratic Republic of the Congo;^11^ these data showed that WHZ alone is more strongly associated with hospital mortality than mid-upper-arm circumference either considered alone or with WHZ after adjustment for age, sex, nutritional oedema, infection and stunting. The presence of nutritional oedema was also found to increase the risk of death.^11^

We observed that conditions that were expected to increase mortality did not appear to be important contributors to death. The presence of acute watery or chronic diarrhoea, dehydration or malaria did not increase the risk of death in children with marasmus. We ascribe these results to affected children receiving effective treatment. Sepsis only became significant after the data were adjusted for confounding factors. The protective association of fever probably served as a marker for children able to mount a beneficial inflammatory reaction.^22^^–^^24^ Although human immunodeficiency virus (HIV) infection is a risk factor for mortality, no data on HIV status were available for this cohort. A recent meta-analysis among children hospitalized with severe acute malnutrition found HIV infection, diarrhoea, pneumonia, shock and lack of appetite were each associated with an increased risk of death.^10^

Among children with oedema, although diarrhoea was not associated with mortality, a diagnosis of dehydration increased mortality. By definition, oedema indicates overhydration; a patient cannot be overhydrated and dehydrated at the same time. Presumably such diagnoses indicate hypovolaemia rather than dehydration. The causes of shock include sepsis, liver failure, cardiogenic shock, toxic shock and drug interactions. Giving excess sodium to overhydrated children with high intracellular sodium concentrations and leaky cell membranes is potentially dangerous.^25^^–^^27^ If there is weight gain (fluid retention) associated with acute respiratory infection, the most likely cause of the tachypnoea is pulmonary oedema secondary to iatrogenic fluid overload leading directly to cardiac failure, not infection. This mechanism would account for the increased mortality associated with both dehydration and tachypnoea in children with oedema without vomiting, diarrhoea or evidence of excess fluid loss. In our results, sepsis is also a purely clinical diagnosis that in reality usually denotes shock or simply severe illness. Most deaths did not occur soon after admission, which could be ascribed to pre-admission factors. As shown in the Kaplan–Meier plots most deaths appeared to occur after the child had deteriorated under treatment in hospital, indicating that some aspect of the treatment may not have been optimal or appropriate.

The finding that older children have a higher risk of death from severe acute malnutrition is consistent with the multicountry pooled study of untreated community cohorts,^28^ which showed a higher mortality for children older than 2 years diagnosed by both mid-upper-arm circumference and WHZ. Although many studies have found younger children to be at higher risk, no significant difference in risk by age and sex was described in one recent meta-analysis.^10^ We expect the proportion of both WHZ-only and older children to be higher in contexts where there is a high caseload of severely malnourished children, such as in severe crisis situations.^29^

Of the children included in this analysis, two thirds were severely stunted. Stunting refers to chronic undernutrition, which has been hypothesized to augment mortality among acutely malnourished children;^28^^,^^30^ our study does not support that conclusion.

Our study was limited by the possibility of confounding from unmeasured factors, as is the case any observational analysis. Nevertheless, the operational nature and the completeness of the data from real-life facilities mean that our data are representative of conditions in low- and middle-income countries, as opposed to experimental studies and trials where additional staff, training and materials are usually temporally available.

Our study benefited from the fact that MSF Operational Centre Paris and the Nigerian health ministry deployed teams trained in WHO inpatient severe acute malnutrition guidelines alongside logisticians, ensuring an uninterrupted supply of therapeutic foods and medicines, clinical supervision and audit via line lists. 

We conclude that mid-upper-arm circumference alone does not identify malnourished children with the highest risk of death; instead, children with a measurement of less than 115 mm had the lowest risk of death. Our inpatient data have shown that children with a low WHZ at admission had twice the risk of death, which increased further if they also had oedema. These children should be subject to special considerations, forming a separate diagnostic category and considered as an exceptionally high-risk group. Moreover, accurate interpretation of signs of dehydration and rapid respiratory rate in children with oedema is particularly important for further mortality reduction. Earlier risk stratification at admission, strengthened diagnostic capacity, standardized shock management (especially for children with oedema) and timely referral systems from outpatient to inpatient care could also improve survival outcomes. 
